# Understanding Curriculum: The Australian Context

**DOI:** 10.1192/pb.bp.114.050351

**Published:** 2016-02

**Authors:** Zaza Lyons

This book is about passion for teaching and learning. The word ‘curriculum’ is used as a metaphor for education at any academic level. The book is composed of 12 chapters containing theoretical background and discussion about specific approaches to the development and delivery of a teaching curriculum, ranging from science and mathematics to fine arts and philosophy. These theoretical discussions focus on both traditional (or conservative) and progressive approaches to education and are accompanied by useful ‘reflective activities’ that help the reader deconstruct and apply underpinning pedagogical concepts and theories to their local context and circumstances. Moreover, each of the chapters contains a set of ‘personal reflections’ that give a human touch to teaching and learning activities in ‘a real life’ setting. Although the book has a focus on the Australian context and curriculum, its messages are universal and applicable to all those who love conveying knowledge or imparting skills to younger generations.

The book also contains an interesting chapter on Indigenous education that focuses on the experiences of a young non-Indigenous teacher who lived and taught in a remote Aboriginal community school in the Northern Territory. Her personal reflections on this unique intercultural encounter and challenging teaching environment make the book relevant to any other multicultural corner of the world. This can be exemplified by the following quote: ‘Understanding multiple ways of knowing becomes increasingly important, as we acknowledge that differing standpoints inform how we human persons think and experience the world’.

Academics involved in medical education rarely have any formal training in teaching. We learn how to teach on the job and in the field and make up for pedagogical shortcomings by an abundance of energy and enthusiasm. Universities around the world are moving towards 4-year graduate medical courses, resulting in a significant reduction in discipline-specific teaching time. With shorter rotations, we need to use our time with students judiciously to ensure that they learn what is needed to cover the core components of the curriculum. Importantly, for an unpopular specialty such as psychiatry, we must also develop more creative and innovative teaching strategies to attract medical students to the ‘endangered’ discipline of psychiatry. The combination of reflective activities and discussion of contemporary educational topics allow this book to serve as a suitable guide for improving psychiatric education that will assist in the survival of psychiatry as a respected career choice and profession.

